# A Modification in the Operation for Hare-lip

**Published:** 1851-10

**Authors:** 


*A modification in the Operation for Hare-lip.*—M. Coste, chief surgeon of the Hôtel Dieu of Marseilles, has been endeavoring to devise the means of obviating the ugly notch which too often remains after the operation for hare-lip. M. Coste states that the modification proposed and practised by M. Malgaigne is only applicable in double hare-lip, and that in the simple deformity M. Malgaigne's method produces an unsightly prominence.

The author in simple hare-lip, (which lies generally on the left side,) has succeeded in avoiding the notch altogether, by cutting a horizontal flap in the red part of the lip on one side, and a kind of half mortise on the other. In paring the margins of the fissure, he takes off more substance than is generally done; the flap and mortise are well secured by twisted suture, and by one of the diminutive spring-forceps called "serre fines;" one transverse needle is placed a little higher up, and no application whatsoever made, so that the progress may be more accurately watched. M. Coste has thus succeeded, upon a little boy, twelve years of age, in completely avoiding the above-mentioned notch.—*London Lancet.*

